# Clinical applications of the mastoid emissary vein

**DOI:** 10.1007/s00276-022-03060-0

**Published:** 2022-12-15

**Authors:** Wei Zhou, Guangfu Di, Jun Rong, Zongwen Hu, Mingze Tan, Kaiqiang Duan, Xiaochun Jiang

**Affiliations:** Department of Neurosurgery, The Translational Research Institute for Neurological Disorders of Wannan Medical College, The First Affiliated Hospital of Wannan Medical College (Yijishan Hospital of Wannan Medical College), Wuhu, 241001 People’s Republic of China

**Keywords:** Mastoid emissary vein, Mastoid foramen, Computed tomography, Craniotomy, Anatomy

## Abstract

**Purpose:**

During retrosigmoid craniotomy, the mastoid emissary vein (MEV) can be a source of considerable bleeding during the operation, especially when the larger diameter MEV or sigmoid sinus is torn. In this study, we evaluated the relevant structure of the MEV for their anatomy and applied the data in surgery to summarize their clinical significance.

**Methods:**

The posterior craniocervical regions of 15 silicon-injected Chinese human cadaver specimens were dissected to expose the MEV and adjacent structures. Fifty-one patients who were scheduled to undergo retrosigmoid craniotomy were selected. All patients underwent preoperative routine CT of the head. The relevant data were collected on cadaveric anatomy and CT. Eventually, all patients underwent retrosigmoid craniotomy and the MEV was observed during the operation.

**Results:**

In cadaver specimens, the prevalence of the MEV was 90.0%. It originated from the middle and lower parts of the posterior wall of the sigmoid sinus and extended in the posterior direction in the mastoid process, usually having 1–2 external openings (86.7%) and only 1 internal opening. The intraosseous courses of the MEV were classified as straight and curved. The straight type accounted for 57.9%, and the curved type for 42.1%. The mean diameter of the MEV was 1.84 ± 0.85 mm, and the straight length of the MEV inside the mastoid process was 11.93 ± 3.58 mm. In 16.7% and 6.7% of all cadaver specimens, the MEV diameter was greater than 2.5 and 4 mm, respectively. In 51 patients (bilateral), routine head CT scan showed the MEV in 49.0% of the patients, and the MEV diameter was greater than 2.5 and 4 mm, respectively, in 17.6% (18/102) and 3.9% (4/102) of the cases. During surgery (unilateral) in the 51 patients, 48 had the MEV and 3 had no MEV. None of the patients had sigmoid sinus tears or massive bleeding.

**Conclusion:**

In the process of retrosigmoid craniotomy, detailed anatomical knowledge of the MEV, well-planned CT scan, and meticulous microsurgical techniques are key for successful operation, which can reduce the occurrence of complications.

## Introduction

The mastoid emissary vein (MEV) is the connection between the sigmoid sinus and the suboccipital venous plexus (SVP), which runs through the mastoid foramen [3, 6, 9, 10]. The sigmoid sinus has been reported to drain into the vertebral plexus in the upright position and into the internal jugular vein in the supine position [3, 8, 10, 12, 17, 21]. Usually, the MEV has no valve structure and the blood flow is slow [2, 6, 9, 16]. However, in patients with intracranial hypertension, internal jugular vein hypoplasia, or aplasia, a dilated MEV is an important channel for cerebral venous drainage and these venous connections may lead to high-flow vascular malformations and become a potential source of uncontrollable bleeding [6, 9, 14, 16].

Retrosigmoid craniotomy is a routine approach for exposing the posterior fossa. The MEV is closely related to the craniotomy process. Sudden and accidental injury to the MEV may lead to hemorrhage. Generally, the MEV is small in diameter, causes very little bleeding, and can be easily controlled using electric coagulation and bone wax. However, when the MEV is large in diameter, the bleeding is very large, especially when the MEV is injured at its confluence into the sigmoid sinus, a sigmoid sinus tear is easily caused, which makes it difficult to control the bleeding and could even lead to shock as well as infection and thrombosis. Kim et al. [8] reported a case of massive hemorrhage caused by injury to the MEV during surgery. Despite the immediate application of pressure, the estimated blood loss in 5 min was higher than 200 ml. Therefore, anatomical variability is valuable not only from an anatomical point of view but also in clinical terms [19]. The success of surgical procedures depends to a large extent on the correct identification and localization of important neurovascular structures, as well as on an adequate preoperative evaluation [11]. Thus far, most previous studies have focused only on the external opening of the MEV, neglecting its complicated intraosseous courses, and have not been integrated with clinical craniotomy [6, 8–10, 14].

Based on these, the purpose of this study is to combine cadaver anatomy with routine CT scan to summarize the anatomical and radiological characteristics of the MEV, and then apply it to the retrosigmoid craniotomy. We believe this can help surgeons better and more accurately understand the significance of the MEV in retrosigmoid craniotomy and reduce the risk of intraoperative bleeding and the occurrence of postoperative complications.

## Materials and methods

### Materials

Fifteen Chinese adult specimens (30 sides) were fixed with 10% formalin. A lack of head trauma and deformity was confirmed in all the specimens. Silicone with red pigments was infused into the arterial system, while silicone with blue pigments was infused into the venous system. All body donors were legally competent and had a will in which they agreed to the use of their body for research, study, or teaching purposes and were provided by the Translational Research Institute for Neurological Disorders of Wannan Medical College. The study was approved by the ethics committee of Wannan Medical College. In all, 51 patients who were admitted to the Department of Neurosurgery of the First Affiliated Hospital (Yijishan Hospital), Wannan Medical College, from September 2017 to December 2021 and planned to undergo retrosigmoid craniotomy were selected. These included 25, 12, 7, 5, and 2 cases of acoustic neurinoma, meningioma, hemifacial spasm, trigeminal neuralgia, and epidermoid cysts, respectively. Written informed consent was obtained from all the patients.

### Cadaveric dissections

In all the wet head specimens, the skin covering the mastoid process and the occipital region was removed to expose the mastoid process and the suboccipital muscle group. The suboccipital muscle group was dissected layer by layer, and each vein structure was preserved. The SVP and its tributaries and the MEV and its openings were fully exposed. First, the number and diameter of the external openings of the MEV and the distance to the asterion and the mastoid tip were measured, and the percentage of MEV diameter greater than 2.5 mm and 4 mm were counted, respectively. Then, partial mastoidectomy was performed to expose the entire length of the MEV, transverse sinus, and sigmoid sinus. The intraosseous courses of the MEV and the number of MEV in the internal opening were observed. The position of the MEV entering the sigmoid sinus was observed, and the distance from the internal opening of the MEV to the transverse sigmoid sinus junction was measured.

### CT scan

All 51 patients (counting bilateral MEV) were evaluated preoperatively by routine CT scan of the head in the bone window mode to visualize the bony anatomy of the posterior cranial fossa. CT of the head was performed using a 16-slice CT scanner (Aquilion). None of the imaging studies were performed for the purposes of this study. The scanning parameters were as follows: slice thickness = 0.6 mm, tube voltage = 120 kVp, tube current range = 300 mA, rotation time = 0.5 s, display field of view (DFOV) = 25 cm × 25 cm, and matrix = 512 × 512. The number and diameter of the external openings of the MEV were measured, and the intraosseous courses of the MEV were observed on CT.

### Clinical applications

All 51 patients (counting unilateral MEV, because the craniotomy was only unilateral) underwent suboccipital retrosigmoid sinus craniotomy. The surgeons were all involved in the cadaveric dissections and had received systematic anatomical training. The MEV was observed during the operation, and instances of intraoperative sigmoid sinus injury and massive hemorrhage were determined.

### Statistical analysis

All the above work, including the analysis of cadaveric dissections, the interpretation of CT images, and the collection of relevant data, was performed by two or more professionals. SPSS for Windows (Version 23) software was used for data analysis and statistical tests. The two-sample *t*-test was used to compare mean values between the two groups. Crosstabulation data were compared between groups by using the chi-square test. A *p*-value < 0.05 was considered statistically different.

## Results

### Cadaver anatomy evaluation of the MEV

**Fig. 1:**
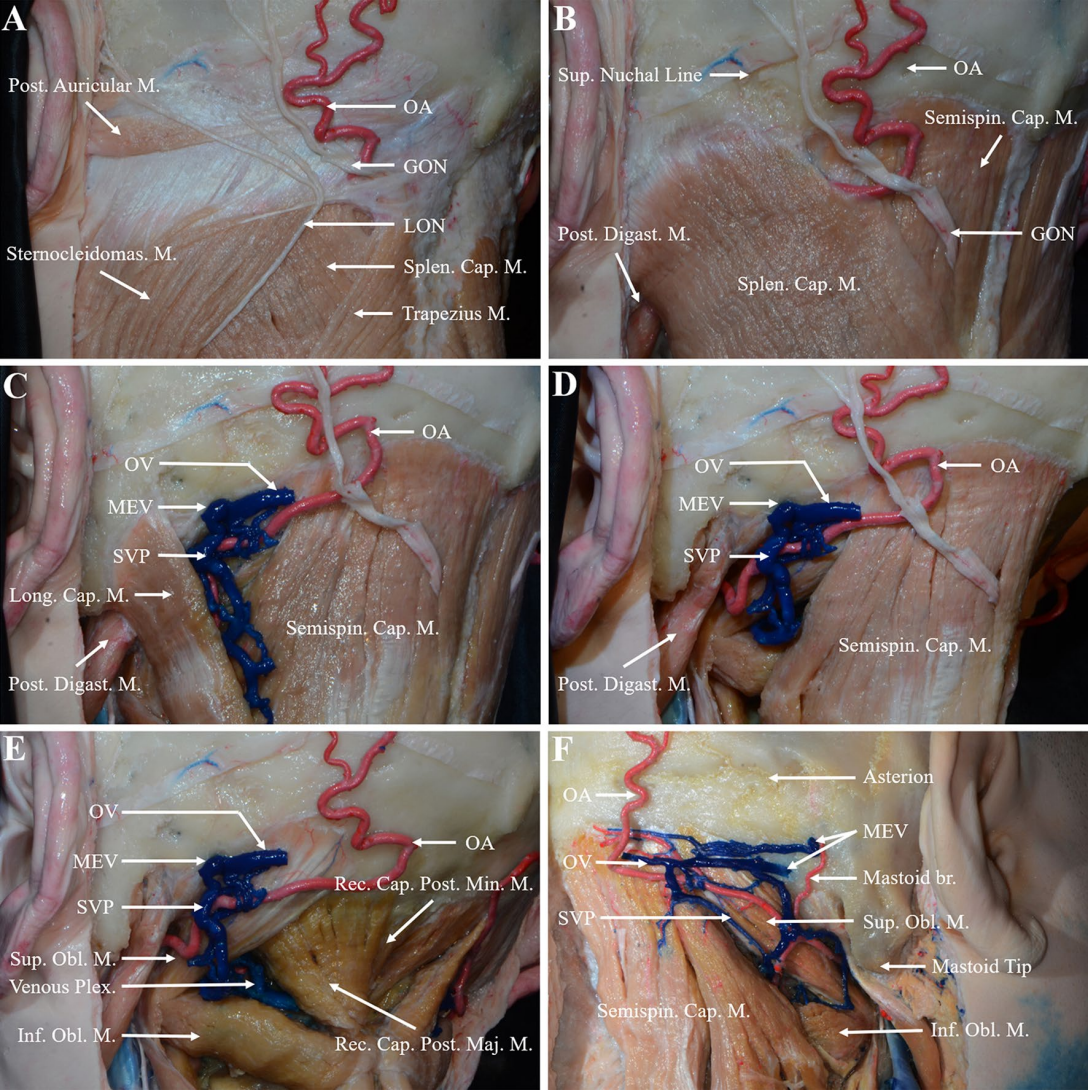
Position of the MEV and SVP in the posterior neck. **A** The occipital skin was excised, and the posterior neck muscles were exposed layer-by-layer. The superficial muscles included the posterior auricular muscle, trapezius muscle, and sternocleidomastoid muscle. The greater occipital nerve intersected with the occipital artery at the medial edge where the sternocleidomastoid muscle was attached. The lesser occipital nerve shallows near the midpoint of the posterior edge of the sternocleidomastoid muscle and then runs upward along the posterior edge of the sternocleidomastoid muscle. **B** The muscles of the middle layer were the semispinalis and splenius capitis muscles. The greater occipital nerve pierced the trapezius muscle and the occipital artery ran deep into the splenius capitis muscle. **C** After the splenius capitis muscle is removed, the SVP was exposed and reached the suboccipital triangle through the cleft between the semispinalis and longissimus capitis muscles. **D** The longissimus capitis muscle was resected to expose the suboccipital triangle. **E** The semispinalis capitis muscle was excised to completely expose the suboccipital triangle, and the SVP was drained through the suboccipital triangle to the venous plexus surrounding the vertebral artery. **F** In the other cadaveric specimen, two MEV were connected to the SVP, and a small artery was shown passing through the mastoid foramen, which was the mastoid branch of the occipital artery and supplied the dura mater of the posterior cranial fossa. *Br*. branch; *Cap*. capitis; *Digast*. digastric; *GON* greater occipital nerve; *Inf*. inferior; *LON* lesser occipital nerve; *Long*. longissimus; *M*. muscle; *Maj*. major; *MEV* mastoid emissary vein; *Min*. minor; *OA* occipital artery; *OV* occipital vein; *Obl*. oblique; *Plex*. plexus; *Post*. posterior; *Rec*. rectus; *Semispin*. semispinalis; *Splen*. splenius; *Sternocleidomas*. sternocleidomastoid; *Sup*. superior; *SVP* suboccipital venous plexus

The MEV originated from the middle and lower parts of the posterior wall of the sigmoid sinus, extended in the posterior direction in the mastoid process, and passed through the mastoid foramen and entered the SVP, which was located in a space between two muscular layers. In all specimens, the splenius capitis muscle formed the roof of this space (Fig. 1B). The longissimus capitis muscle was located on the lateral side, and the semispinalis capitis muscle was located on the medial side and formed the floor (Fig. 1C). The SVP reached an inner muscular layer through the muscular cleft between these two muscles. In this layer, the floor was the suboccipital triangle, which was formed by the superior and inferior oblique muscles and the rectus capitis posterior major muscle. Then SVP drained through the suboccipital triangle to the venous plexus surrounding the vertebral artery (Fig. 1D, E).

**Fig. 2:**
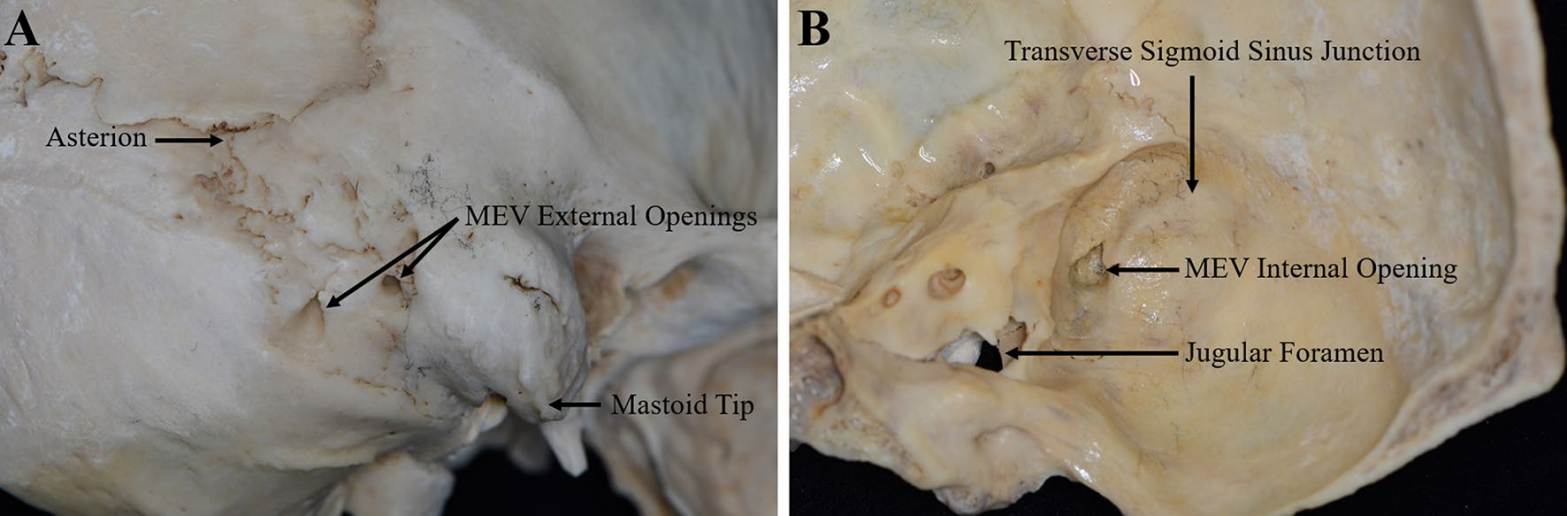
Dry skull specimens show the locations of the external and internal opening of the MEV. **A** The position of the external opening of the MEV; the distance from each external opening to the asterion and the mastoid tip was measured. **B** The position of the internal opening of the MEV; the distance from each internal opening of the MEV to the transverse sigmoid sinus junction was measured

**Fig. 3:**
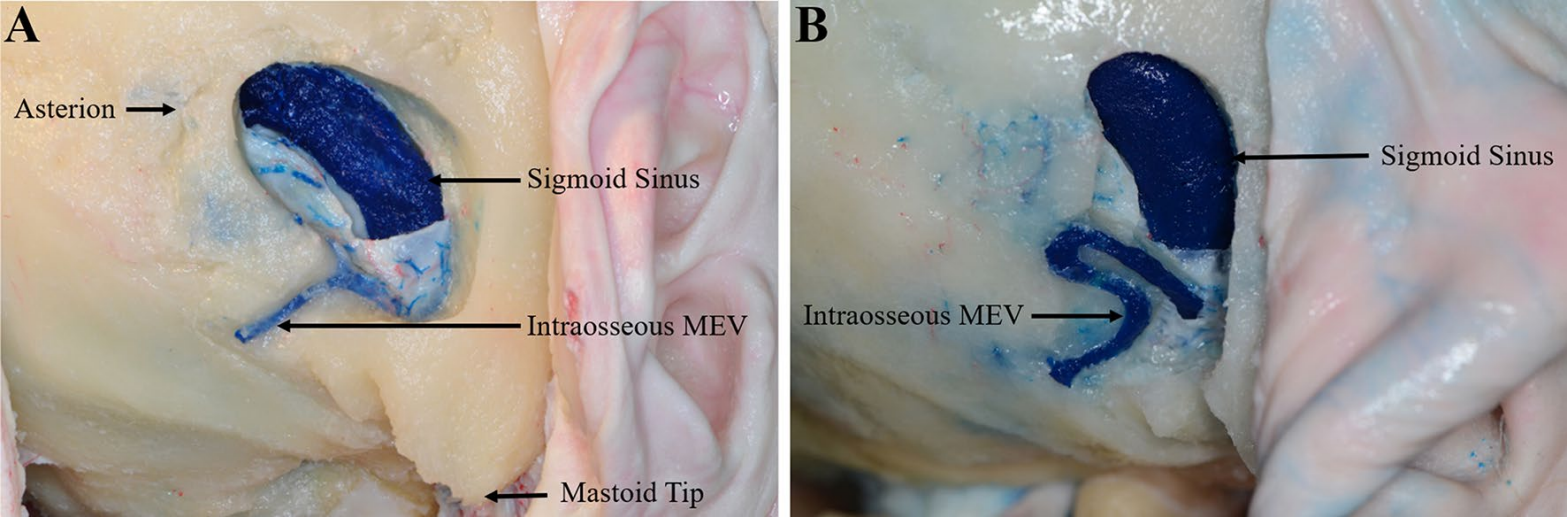
The intraosseous courses of the MEV on cadaveric specimens. **A** Straight type. **B** Curved type

The MEV was found in 90.0% (27/30) of all 15 cadaver specimens. One external opening was the most common, accounting for 56.7% (17/30) of the cases, followed by two and three external openings in 30% (9/30) and 3.3% (1/30) of the cases, respectively, for a total of 38 external openings. Only one internal opening was found in all 15 cadaver specimens with the MEV, and 10.0% (3/30) had no internal or external opening. The mean distance between the asterion and the mastoid tip was 47.17 ± 3.98 mm (range, 41.59–57.18 mm). The external opening was located a mean of 22.33 ± 4.94 mm (range, 13.56–31.74 mm) from the asterion and a mean of 28.23 ± 4.02 mm (range, 18.82–37.24 mm) from the mastoid tip. The mean diameter of the MEV at the external openings was 1.84 ± 0.85 mm (range, 0.78–4.57 mm), and the straight length of the MEV inside the mastoid process was 11.93 ± 3.58 mm (range, 6.45–19.32 mm). In 16.7% (5/30) and 6.7% (2/30) of all cadaver specimens, the MEV diameter was greater than 2.5 and 4 mm, respectively (Fig. 2A). The intraosseous courses of the MEV were classified as straight or curved, with the straight and curved types accounting for 57.9% (23/38) and 42.1% (15/38) of the cases, respectively (Fig. 3A, B). The mean distance from the internal opening to the transverse sigmoid sinus junction was 17.35 ± 2.9 mm (range, 12.58–22.57 mm) (Fig. 2B).

### The MEV on CT scan

**Fig. 4:**
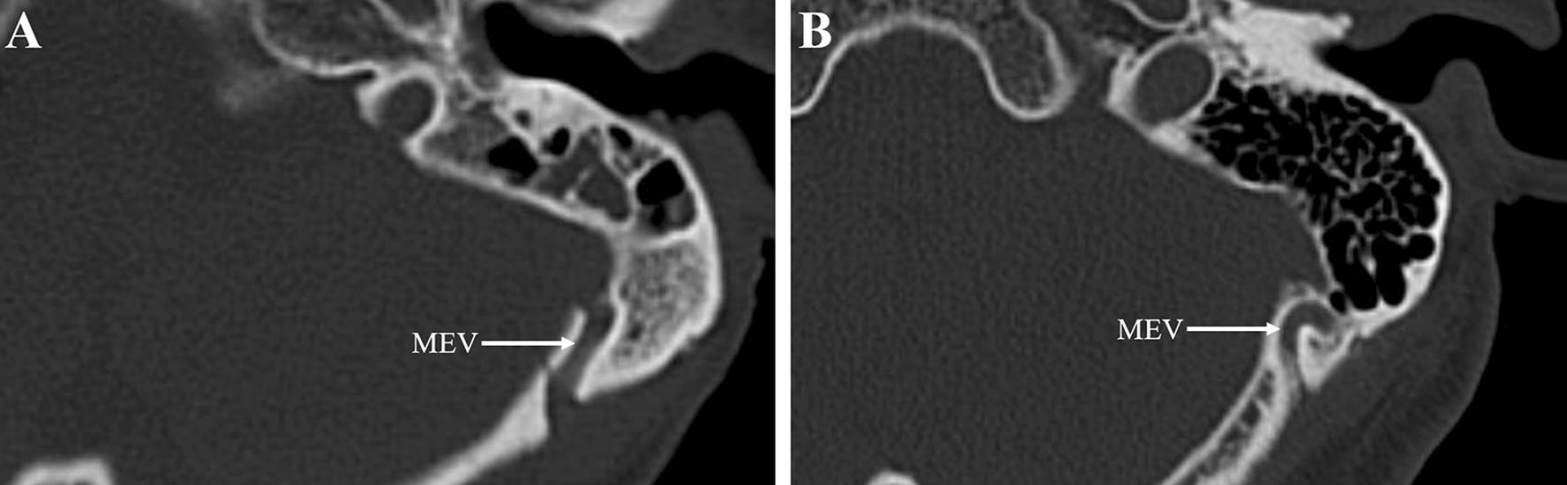
The MEV on CT bone window mode. **A** Straight type. **B** Curved type

The MEV was found in 49.0% (50/102) of all 51 patients (bilateral) on CT scan, with a total of 63 external openings. The mean diameter of the MEV at the external openings was 2.06 ± 1.03 mm (range, 0.70–5.61 mm), and the MEV diameter was greater than 2.5 and 4 mm, respectively, in 17.6% (18/102) and 3.9% (4/102) of the cases. The straight and curved types of MEV accounted for 49.2% (31/63) and 50.8% (32/63) of cases, respectively (Fig. 4). There was a statistically significant difference in the incidence of MEV on conventional CT scans compared to cadaveric dissections, but no significant difference in the detection of MEV larger than 2.5 mm and 4 mm in diameter (Table 1).

**Table 1:** Comparison of the prevalence, mean diameter, and intraosseous course of the MEV between cadaveric dissections and CT

	Cadaveric dissections	CT	*p*-value
Prevalence	90.0% (27/30)	49.0% (50/102)	0.000
Mean diameter (mm)	1.84	2.06	0.285
> 2.5	16.7% (5/30)	17.6% (18/102)	0.901
> 4	6.7% (2/30)	3.9% (4/102)	0.526
Intraosseous course			
Straight type	57.9% (23/38)	49.2% (31/63)	0.269
Curved type	42.1% (15/38)	50.8% (32/63)	

### Intraoperative situation of the MEV

**Fig. 5:**
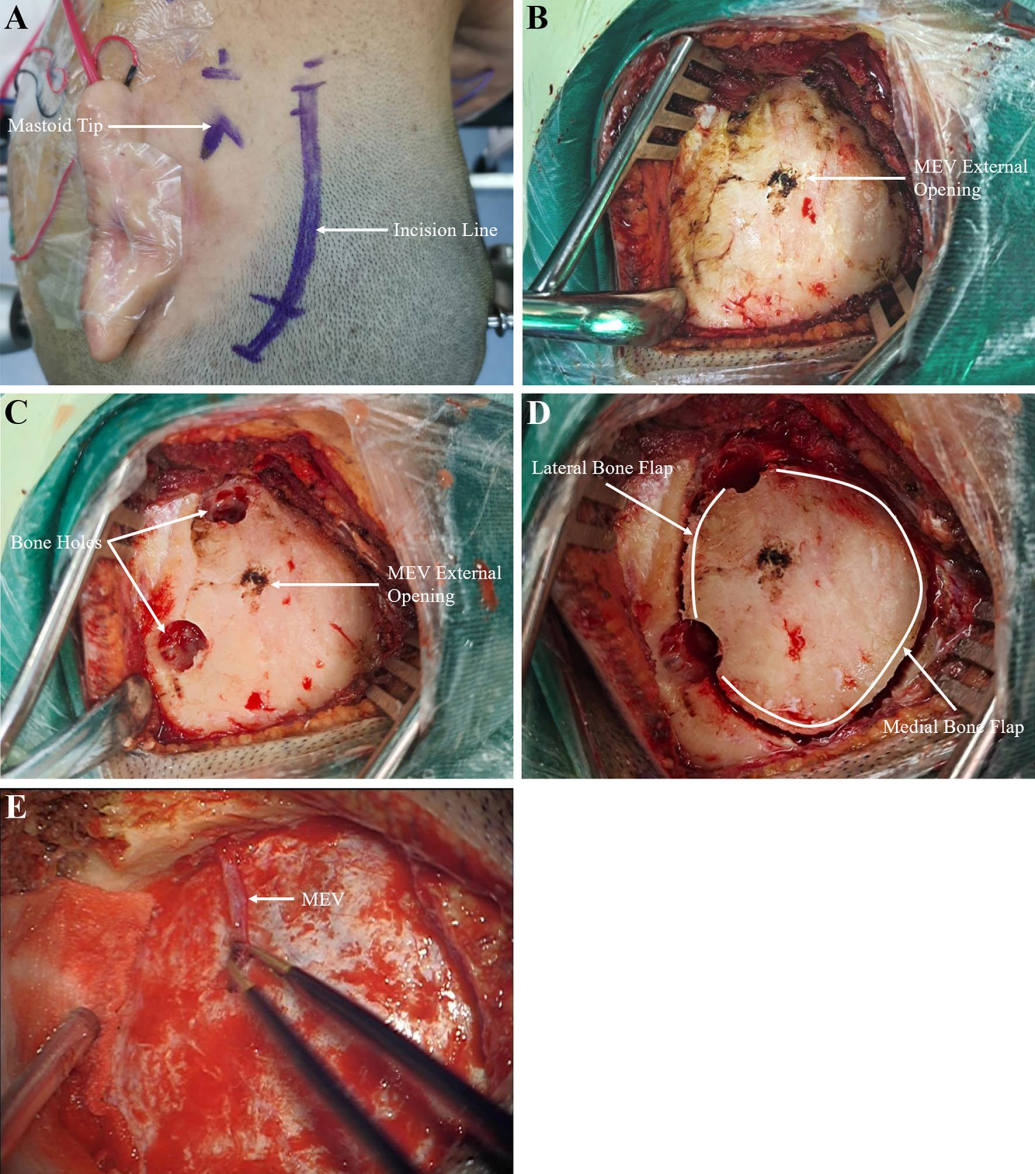
Intraoperative situation related to the MEV. **A** The incision line of the retrosigmoid approach was drawn, showing an arc incision passing through the asterion. **B** The scalp and muscle were cut open to reveal the bone flap. At the external opening of the MEV, electrocoagulation and burning combined with bone wax filling for hemostasis were performed. **C** Two bone holes were drilled. **D** The bone flap was removed with a milling cutter. **E** The full length of the MEV was exposed without sigmoid sinus laceration and massive bleeding after the bone flap was removed

**Fig. 6:**
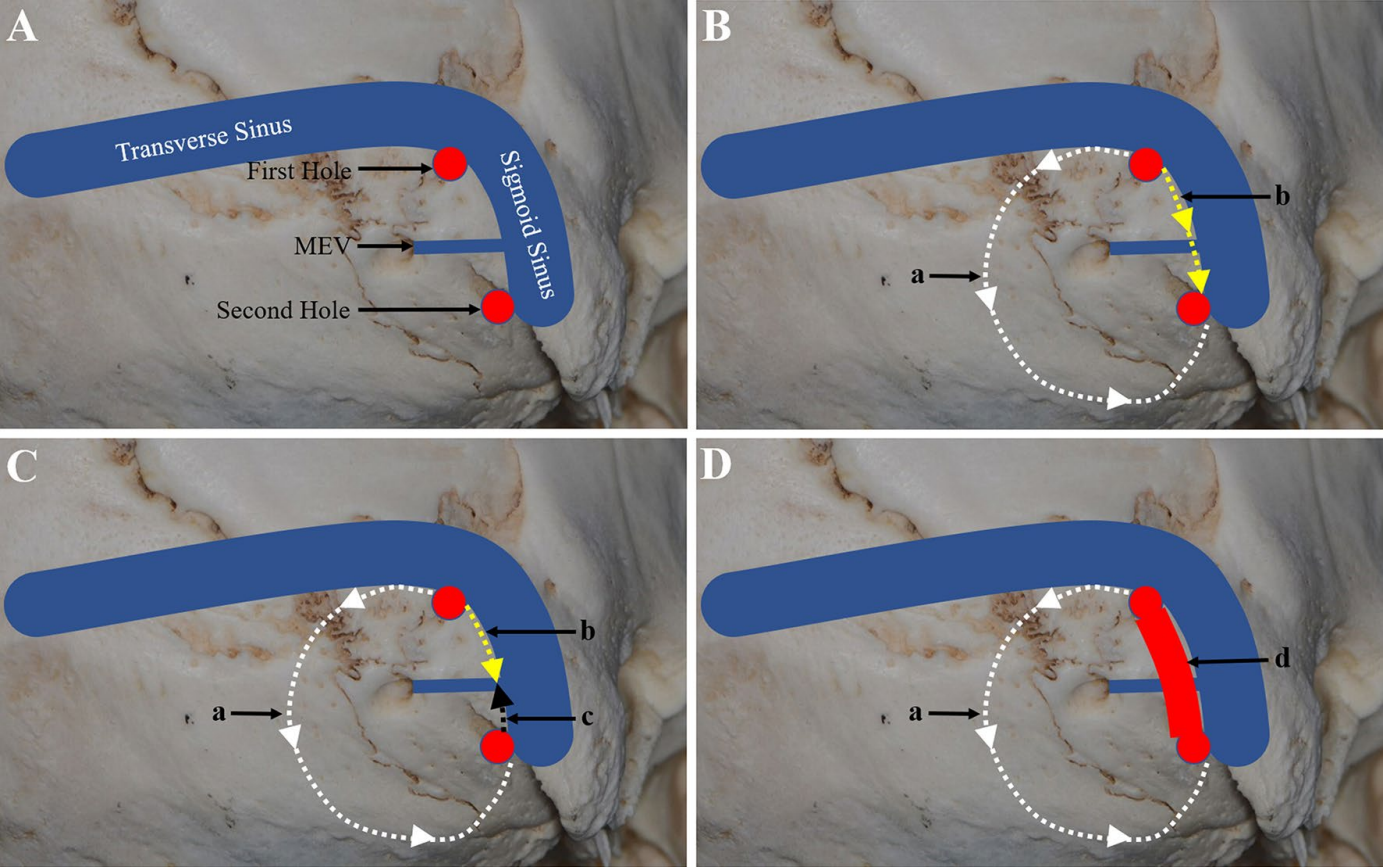
Schematic diagram of MEV treatment at the internal opening. **A** Two bone holes were drilled. One bone hole was located at the transverse sigmoid sinus junction (First Hole), and the other was located at the outermost edge of the exposed bone flap and above the sigmoid sinus entering the jugular bulb (Second Hole). **B** The medial bone flap can be milled directly with a milling cutter (a), and the lateral bone flap can be milled from the top to the bottom with a milling cutter (b). In the absence of bleeding, the flap can be milled directly. **C** In cases showing bleeding, the milling is stopped, the milling cutter is taken out, and the remaining bone flap is milled from the bottom to the top to connect the whole bone flap (c). **D** When the diameter of the MEV is larger (> 4 mm) as indicated by preoperative CT, the lateral bone flap should be carefully ground and removed (d), fully exposing the MEV and protecting the integrity of the junction between the MEV and sigmoid sinus. First Hole and Second Hole, red circles; a, white dashed line with arrow; b, yellow dashed line with arrow; c, black dashed line with arrow; d, red arch (colour figure online)

Among the 51 patients (unilateral), 48 patients had the MEV and 3 did not. No sigmoid sinus tear or massive hemorrhage was noted during the operation (Figs. 5, 6).

## Discussion

The MEV connects the sigmoid sinus with the SVP, and its prevalence, diameter and course vary greatly. The MEV has been reported to be an important source of bleeding in retrosigmoid approach surgery [1, 6, 9, 10, 16]. Therefore, surgeons need to be familiar with its structure.

### Anatomical evaluation of the MEV

Previous data from different populations had a higher prevalence of specimens having the MEV. Murlimanju et al. [10] reported that the prevalence of mastoid foramen was 91.7% in 88 temporal bones, with one mastoid foramen in 62.5%, two mastoid foramens in 22.9%, three mastoid foramens in 6.2%, and no mastoid foramen in 8.3%. Louis et al. [9] noted that the prevalence of the mastoid foramen was 98% and 72% on the right and left sides of the skull, respectively. Kim et al. [8] found that 83.7% of 106 skulls examined had at least one mastoid foramen. Reis et al. [14] examined 14 human cadavers and found that 89% of the skulls had the MEV. In the present study, cadaveric specimens were found to have a high prevalence of MEV (90%), similar to previous studies.

The diameter of the MEV varies greatly. Louis et al. [9] reported that the mean diameter of MEV at the external opening was 3.5 mm (range, 1.1–5.6 mm), and there was no statistically significant correlation between the type of foramen and the diameter or length. Kim et al. [8] stated that if the diameter of the MEV was greater than 2.5 mm, the vessel can be considered to be sufficiently large in diameter and size to require dissection and definition before formal ligation, rather than being simply controlled by electrocoagulation. They reported a mean diameter of 1.64 mm for the mastoid foramen. In 15.0% of the skull specimens, the diameter of the MEV foramen was greater than 2.5 mm, while in 4.3% of the specimens, the diameter was > 4 mm [8]. Forte et al. [4] observed 115 skull specimens and found that 60.0% of the MEV were less than 2 mm in diameter, 25.0% were between 2–3.5 mm, and 15.0% were greater than 3.5 mm. Hampl et al. [6] reported the findings for 295 skulls, in which the mean size of the MEV at the external opening was 1.3 mm (range, 0–7.3 mm), and the mean size at the internal opening was 1.7 mm (range, 0.2–9.0 mm). This study showed that the mean diameter of the MEV was 1.84 ± 0.85 mm, with 16.7% of cadaveric specimens showing the MEV > 2.5 mm in diameter and 6.7% showing > 4 mm in diameter. Simultaneously, we found that the mastoid foramen not only included the MEV but also may pass through a small artery, which was the mastoid branch of the occipital artery and supplied the dura mater of the posterior cranial fossa, which may have led previous researchers to overestimate the directly measured diameter of the MEV in skull specimens (Fig. 1F). In addition, previous studies have shown that a larger MEV was associated with smaller jugular foramen, and this larger MEV can drain the transverse sinus or sigmoid sinus to the occipital vein and further to the external jugular vein system or the vertebral vein system [18, 22].

The MEV also varied greatly as it traveled through the mastoid canal. In this study, 42.1% of the MEV traveled tortuously in the mastoid canal, and part of the MEV was connected to the diploic vein. In craniotomy involving the MEV, the curved MEV was prone to injury and led to bleed. Therefore, a thorough understanding of the preoperative course of the MEV is important, and CT scan can achieve this objective well (Fig. 4).

### Radiological study of the MEV

The MEV can be shown on magnetic resonance imaging (MRI), CT, and angiography. Pekcevik et al. [13] considered that CT angiography was a valuable tool for assessing the emissary veins and venous vascular canals. The MEV was delineated in 77.7% of the patients, which were superior to MR venography in depicting venous structures with slower flow and smaller diameters. It also has the advantage of imaging the bony canal. Tsutsumi et al. [20] suggested that MRI may be a reliable method for detecting the MEV, since it delineated the MEV in 89.5% of patients, which were a better result than that in a previous report that used CT angiography and similar to the results of studies with cadaveric specimens. Roser et al. [16] compared standard CT (4.5 mm) with thin-slice CT (1 mm). Standard CT revealed a mean of 0.3 MEV on the right side and 0.5 MEV on the left side, while thin-slice CT revealed a mean of 1.6 MEV on the right side and 1.7 MEV on the left side. These findings were significantly different. Therefore, they concluded that thin-slice CT is critical in preoperative planning of the posterior fossa approaches to identify the presence and course of MEV [16]. In our study, routine CT scan of the head showed the MEV in only 49.0% of patients, which apparently lacked the sensitivity to detect some of the smaller MEV, resulting in an overall MEV prevalence much lower than that reported in our cadaveric specimen study and by others. However, there is no significant difference between conventional CT in detecting MEV larger than 2.5 mm in diameter compared to cadaveric specimens, and since the main point of our surgical concern is the larger diameter MEV, it can be easily resolved for smaller diameter MEV or no MEV. Therefore, preoperative routine CT examinations are sufficient to understand the structures associated with the MEV (include the original sites on the sigmoid sinus wall, sizes, and intraosseous courses of the MEV), reducing both the amount of radiation exposure and cost to the patient compared to previous studies. Thus, this study suggests that preoperative routine CT scan of the MEV is essential for surgical procedures involving the mastoid process region to identify the presence and course of larger diameter MEV, thereby reduce the preventable intraoperative complications associated with the approach.

### Significance of the MEV during the operation

The standard incision in the retrosigmoid approach begins in the posterior region of the mastoid process, passes through the asterion, and enters the lateral part of the posterior neck downward (Fig. 5A). Its trajectory is consistent with the anatomical position of the superficial neck vein system, which is prone to cause massive bleeding in the muscle anatomy. Reis et al. [14] stated that the ideal incision line in the retrosigmoid approach should be made 4–5 cm medial to the mastoid process to prevent damage to vascular structures in the mastoid process and all its consequences.

Second, considering the short course of the MEV and emergency from the smooth surface of the posterior mastoid process, injury to the MEV typically occurs at the external opening and is difficult to dissect and ligate. Our experience is that direct electrocoagulation and cauterization combined with bone wax filling can stop bleeding at the external opening of MEV (Fig. 5B). However, bone wax may migrate to the sigmoid sinus, leading to venous sinus thrombosis. Postoperative monitoring of neurological function should be performed carefully. Hadeishi et al. [5] reported that in 161 patients undergoing retromastoid craniotomy, bone wax migrated to the sigmoid sinus in 7 patients, all of whom had a large mastoid foramen and required a large amount of bone wax to control bleeding during retromastoid craniotomy. Therefore, when the diameter of MEV is larger (> 4 mm), as indicated by preoperative CT, ligation should be performed in advance in addition to the use of bone wax.

Moreover, at the internal opening of MEV, the tear of the sigmoid sinus is another important source of massive hemorrhage. Our experience involved drilling two-bone holes. One hole was located at the transverse sigmoid sinus junction, and the other was located at the outermost edge of the exposed bone flap and above the sigmoid sinus entering the jugular bulb (Fig. 6A). First, the medial bone flap was milled with a milling cutter, after which the lateral bone flap could be milled from top to bottom with a milling cutter (Fig. 6B). In cases involving bleeding, the procedure is stopped, the milling cutter is taken out, and the remaining bone flap is milled from the bottom to the top to connect the whole bone flap so as to minimize the possibility of a sigmoid sinus tear (Fig. 6C). However, when the diameter of the MEV is larger (> 4 mm), as indicated by preoperative CT, the lateral bone flap should be carefully grinded and removed to fully expose the MEV, protect the integrity of the junction between the MEV and sigmoid sinus, and reduce the occurrence of bleeding (Fig. 6D). We reported the findings for 51 cases of craniotomy via the retrosigmoid approach. The patients showed was no sigmoid sinus tear and massive hemorrhage during the operation. Some authors have reported that the MEV may be the main outflow pathway of the posterior fossa dural sinuses in some cases, and ligation may lead to venous ischemia and hemorrhage [15]. Hoshi et al. [7] reported two cases of cerebellar infarction and one death resulting from coagulation of the MEV during skull-base surgery. There was no case of cerebral infarction caused by MEV occlusion in this study.

#### Strengths and limitations of the study

The strengths of the present study were that it was a combination of cadaver anatomy and conventional CT scan to summarize the anatomical and radiological characteristics of the MEV, which can help surgeons to better and more accurately understand the significance of MEV in retrosigmoid craniotomy and reduce the risk of intraoperative bleeding and postoperative complications. The limitations were the small number of cadaveric specimens and clinical cases, which may affect the final experimental results. Second, the effects of the right and left sides and gender was not further distinguished. Therefore, further large-scale studies are needed.

## Conclusion

Familiarity with the anatomical structure of the MEV can help surgeons avoid or address the vascular bleeding caused by trauma in advance. Head CT scan can help surgeons understand the diameter and course of the MEV to formulate the surgical strategy in advance. Meticulous microsurgical skills can prevent the occurrence of potentially fatal complications.

## Data Availability

The data that support the findings of this study are available from the corresponding author upon reasonable request.
